# Sparsity and Compressed Coding in Sensory Systems

**DOI:** 10.1371/journal.pcbi.1003793

**Published:** 2014-08-21

**Authors:** Victor J. Barranca, Gregor Kovačič, Douglas Zhou, David Cai

**Affiliations:** 1 Courant Institute of Mathematical Sciences and Center for Neural Science, New York University, New York, New York, United States of America; 2 NYUAD Institute, New York University Abu Dhabi, Abu Dhabi, United Arab Emirates; 3 Mathematical Sciences Department, Rensselaer Polytechnic Institute, Troy, New York, United States of America; 4 Department of Mathematics, MOE-LSC, and Institute of Natural Sciences, Shanghai Jiao Tong University, Shanghai, China; Indiana University, United States of America

## Abstract

Considering that many natural stimuli are sparse, can a sensory system evolve to take advantage of this sparsity? We explore this question and show that significant downstream reductions in the numbers of neurons transmitting stimuli observed in early sensory pathways might be a consequence of this sparsity. First, we model an early sensory pathway using an idealized neuronal network comprised of receptors and downstream sensory neurons. Then, by revealing a linear structure intrinsic to neuronal network dynamics, our work points to a potential mechanism for transmitting sparse stimuli, related to compressed-sensing (CS) type data acquisition. Through simulation, we examine the characteristics of networks that are optimal in sparsity encoding, and the impact of localized receptive fields beyond conventional CS theory. The results of this work suggest a new network framework of signal sparsity, freeing the notion from any dependence on specific component-space representations. We expect our CS network mechanism to provide guidance for studying sparse stimulus transmission along realistic sensory pathways as well as engineering network designs that utilize sparsity encoding.

## Author Summary

In forming a mental percept of the surrounding world, sensory information is processed and transmitted through a wide array of neuronal networks of various sizes and functionalities. Despite, and perhaps because of, this, sensory systems are able to render highly accurate representations of stimuli. In the retina, for example, photoreceptors transform light into electric signals, which are later processed by a significantly smaller network of ganglion cells before entering the optic nerve. How then is sensory information preserved along such a pathway? In this work, we put forth a possible answer to this question using compressed sensing, a recent advance in the field of signal processing that demonstrates how sparse signals can be reconstructed using very few samples. Through model simulation, we discover that stimuli can be recovered from ganglion-cell dynamics, and demonstrate how localized receptive fields improve stimulus encoding. We hypothesize that organisms have evolved to utilize the sparsity of stimuli, demonstrating that compressed sensing may be a universal information-processing framework underlying both information acquisition and retention in sensory systems.

## Introduction

It is well known that natural stimuli, such as visual images, are sparse in the sense that they can be well represented by a small number of dominant components, typically in an appropriate frequency space [1]. We may thus naturally expect that organisms' sensing has evolved to be adapted to such sparsity. One sign of this adaptation may be the great reduction in numbers between the receptor cells and the sensory neurons in the immediate downstream layers along the early stages of sensory pathways [2], [3]. For example, in the retina, the stimuli received by ∼150 million rods and cones are transmitted through only ∼1.5 million retinal ganglion cells [2]. More generally, it is important to know how the network topology of early sensory pathways reflects this type of adaptation. How have the networks along these pathways evolved so that they can best transmit sparse stimuli and the least amount of information is lost through network dynamics [4], [5]?

Theoretically, the above question translates into the search for a class of neuronal networks that takes advantage of stimulus sparsity and thus best encodes such stimuli. Naturally, such networks should need relatively few downstream neurons to sample the input from the receptors. An instructive technological analog is provided by the *compressed sensing* (CS) theory [6], [7]. When using sufficiently random sampling of sparse images, this theory allows us to dramatically reduce the sampling rate as compared to that expected for the uniform sampling of finite-bandwidth stimuli [8], without degrading the image reconstruction. Greatly improving the fidelity of high dimensional data reconstructions and developing efficient sampling algorithms, applications of CS have emerged in numerous fields, including physics, biology, and imaging [9]–[13].

In the context of neuroscience, it has been conjectured that the information processing in the brain may be related to the existence of an efficient coding scheme, such as compressed sensing [14], [15]. Using adaptive CS, for example, sparse representations of sets of sub-sampled inputs can be developed through unsupervised learning without knowledge of either the sampling protocol or the sparse basis of the measured signal, revealing that CS may, in theory, help to explain signal interpretation and transmission in the brain [16]. Following the CS mathematical structure, it has also been suggested that linear, discrete-time network dynamics can be used to encode sparse temporal sequences of information in their current activity and therefore neuronal networks may possess a greater theoretical memory capacity than previously hypothesized [17]. In this work, we take a new direction by constructing a spiking-neuron network model of an early sensory pathway and demonstrating how the firing rates of a relatively small set of sensory neurons with nonlinear dynamics can successfully encode network inputs. Deriving a linear mapping embedded in the network dynamics, we use CS theory and the dynamics of our model network over a biologically realistic time-scale to reconstruct visual stimuli, which are known to be sparse in frequency space [1]. We also find that the performance of this model can be greatly improved by incorporating the biologically realistic property of localized receptive fields [18], [19]. Unlike previous work [14], [15], the derived input-output relationship is not constructed through learning, and is instead intrinsic to the network dynamics, suggesting a possible way sensory information is transmitted downstream via sparse coding of stimuli through network dynamics.

Even before the discovery of CS, sparse coding was hypothesized as a feature fundamental to optimally representing sensory information, thus possibly leading to the emergence of spatial receptive-field properties of simple cells in the primary visual cortex [20], [21]. Instead of using the framework of optimization [20], [21], we consider how the time-evolving output of populations of firing neurons encodes stimulus information and examine the key characteristics of a CS neuronal network best evolved to transmit sparse stimuli. Underlining a novel notion of sparsity in terms of network dynamics, our results suggest a stimulus may be considered sparse if it can be accurately encoded by networks in which the number of downstream neurons is much lower than the number of input components, separating the notion of sparsity from any dependence on a particular component-space transform choice.

## Results

### Minimal Compressed Sensing Network

To study sparse stimulus transmission along early stages of sensory pathways, we have constructed our conceptual network model to consist of two layers, an input layer and a processing layer, representing the receptors and sensory neurons (sensory cells), e.g., retinal ganglion cells in the retina. Invoking the fact that the receptor neurons in the retina exhibit graded-potential rather than the usual action-potential responses [22], we represent the input layer by currents injected into the sensory neurons in the processing layer. Each current represents the stimulus intensity in the receptive field of a given receptor, which relays this intensity downstream to a number of sensory neurons. We describe these neurons using the pulse-coupled, integrate-and-fire (I&F) model [23]–[30]. Our model is intentionally idealized, so that only the most general features of early sensory pathways are incorporated. In this way, we aim to emphasize the possible role of the CS mechanism in a broad class of sensory systems.

In our model, the membrane-potential dynamics of the 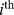 sensory neuron is governed by the differential equation 
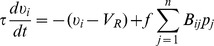


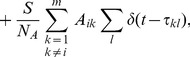
(1)and evolves from the reset potential 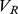 until it reaches the threshold potential 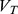. At the 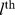 time this occurs, 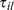, we say that this neuron has fired (or spiked), reset 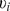 to 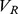, and inject the currents 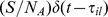 into all the other sensory neurons post-connected to it, with 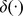 being the Dirac delta function. Here, 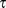 is the membrane-potential time-scale, 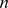 and 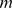 are the numbers of the receptors and sensory neurons, respectively, 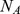 is the number of sensory-neuron connections, 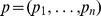 are the stimulus strengths transmitted by the receptors, 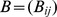 and 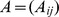 are connection matrices between the receptors and sensory neurons and between sensory neuron pairs, respectively, and 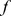 and 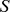 are the respective overall strengths of those connections. Stimulus components, 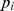, take on integer values between 0 and 255, indicating the light intensity of the stimulus. These components will typically be fixed over time, since we primarily consider stationary stimuli. We simulate this model for a run-time of 200 ms using an event-driven algorithm in which we analytically solve for sensory neuron voltages and spike times, choosing parameters 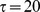 ms, the dimensionless potential values 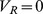 and 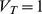, 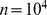, 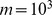, 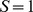, and 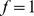 [31], [32].

We first assume that every sensory neuron samples the stimulus randomly from the entire receptor pool, and choose the numbers 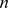 of the receptors and 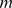 of the sensory neurons to be such that 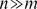 (In most of our computations, due to the limitations imposed by our computational power, we take the ratio *n*∶*m* to be 10∶1 instead of the 100∶1 observed in early sensory pathways [2], [3].) While this assumption of random sampling is fundamental to conventional CS theory, we later consider the more realistic case in which photoreceptors are sampled locally by sensory neurons, which yields a significant improvement in stimulus encoding [18], [19]. Moreover, the sensory neurons are also initially assumed to be connected to each other randomly, but, as we will subsequently demonstrate, we can also assume that the sensory neurons are uncoupled without affecting the results of this work. While retinal ganglion cells, for example, are in some cases not thought to be connected to each other, there are also other cases in which connectivity is observed, and we therefore address this by considering both scenarios [33]–[35]. Although ganglion-cell connections are typically gap junctions [34], we model these connections with pulse-coupling to maintain model idealization and simplicity. Therefore, we first take the elements 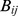 and 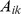 of both connectivity matrices to be Bernoulli-distributed, and later consider several realistic alternative assumptions, such as the localized properties of receptive fields [36]. The inputs into the sensory neurons are assumed noiseless in our preliminary discussion, but we will address the impact of more biological noisy processing, due to fluctuations in photon absorption for example, in the upcoming section [37].

We emphasize that we are modeling a general early sensory pathway, rather than incorporating details specific to the retina, and therefore omit several detailed properties in order to accentuate the underlying CS mechanism. For example, compared to the actual retinal network, we only consider “on,” rod-like receptors, neglecting any time-course details of the graded potentials the receptors produce [38] and any crosstalk among the receptors [39]. In addition, we also neglect the rich variety of the neuron types in the retina [40] and their complex connectivity [41], the center-surround structure of the ganglion neurons' receptive fields [18], [19], any spatial differences in the density of the receptor distribution [42], as well as any inhibition [22].

**Figure 1:**
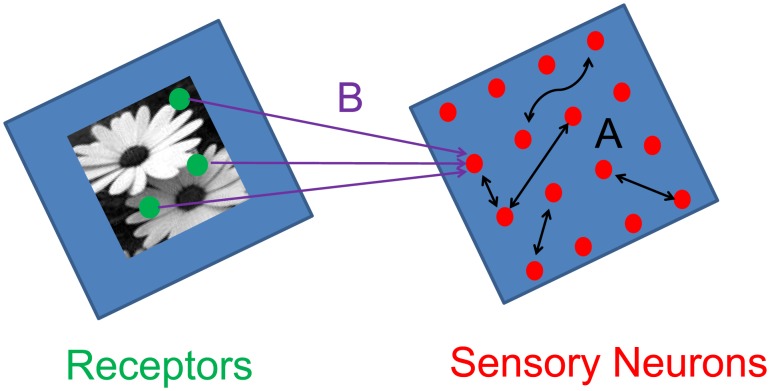
Mathematical model. Graphical depiction of mathematical model and network connectivity.

To determine the degree of connectivity between our networks, we introduce the notion of convergence, which is defined as the average number of neurons presynaptic to any neuron in a given network. In particular, we use a convergence of 50 for 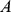, the sensory-neuron connection matrix, and a convergence of 10 for 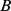, the sensory-neuron to receptor connection matrix. The architecture of the network is represented graphically in Fig. 1. For this neuronal network, conceptually, the question is whether its dynamics take advantage of the sparse stimulus structure, and whether its topology can effectively and efficiently transduce the input information to the sensory neurons.

The above question translates to how to design the network parameters so that the information from the original stimulus is best retained by the firing rates embedded in sensory-neuron spike trains when 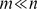, i.e., how closely we can reconstruct the original stimulus from the sensory neurons' firing rates given the model network's connectivity. A stimulus presented to 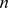 receptors is considered 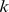-sparse, with 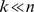, when at least one of its transforms into an appropriate frequency or wavenumber space, such as Fourier or wavelet, has at most 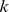 components whose magnitude exceeds a small threshold 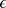 [6], [43]. Given such a stimulus, our model sensory-neuron network generates a set of spike trains, which presumably encodes sparse stimulus information.

If we want to use the CS theory as a guiding principle in our model network construction, we immediately encounter a conceptual difficulty because a prerequisite for CS is linear signal measurement, whereas neuronal dynamics are nonlinear. However, it turns out that there is a linear structure corresponding to the input-output relationship embedded within this network. Using coarse-graining methods similar to kinetic theory in nonequilibrium statistical physics, we derive the linearized firing-rate system 
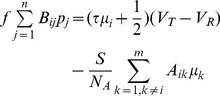
(2)valid when the neuronal firing rates, 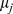, satisfy 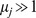 for all 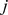 and the membrane potential jump induced by each spike is small, 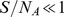 [44], [45]. The firing-rate model (2) was previously derived in the population sense for ensembles of neuronal networks with stochastic inputs of homogeneous strength. However, our work here reveals that through coarse-graining over an ensemble of network realizations differing in initial voltage conditions, in which each network realization is forced by the same set of heterogeneous deterministic inputs, this firing-rate model can be extended to each individual neuron coupled in the network. For weak sensory-neuron coupling-strength and high sensory-neuron firing rates, we therefore obtain a linear network mapping of the stimulus intensities 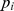 arriving at each receptor onto the firing rates 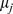 generated by each sensory neuron. In this case, the network is mean-driven, with each sensory neuron receiving a large number of small inputs from its neighbors, which can be approximated by a Poisson spike train of inputs. Under this assumption, we derive a non-linear input-output mapping, which we then linearize in the 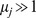 limit. The linear network mapping (2) is between the 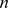-dimensional input vector 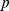 and the 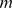-dimensional output vector of neuronal firing rates 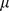; it is not a map between population-averaged input (the network input) and the population-averaged output (the network firing rate) as in traditional coarse-graining applications [44], [45].

**Figure 2:**
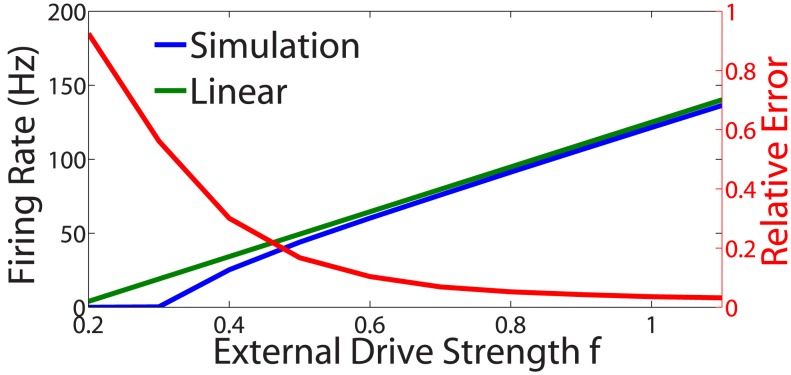
Validity of network firing-rate mapping. Closeness of the firing rates produced by the I&F model and firing-rate mapping for each neuron in the network. The stimulus used is the image in Fig. 3c; Left ordinate axis: Gain curves depicting the dependence of the *network-averaged* firing rate on the overall external drive strength. Right ordinate axis: The same dependence of the cumulative neuron-to-neuron relative firing rate difference between the I&F model and the linear network mapping. This difference is 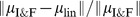, where 
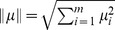
, and 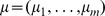 represents the vector of the sensory-neuron firing rates.

The proximity of the firing rates we have obtained from the I&F model (1) and the linear network mapping (2) is depicted in Fig. 2. The red line displays the dependence of the relative firing rate difference on the overall stimulus intensity 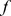. Since the firing rate of each neuron is determined by its unique external input and network connectivity, the error given in Fig. 2 is the sum of the errors for all individual neurons. It is clear that, *neuron-by-neuron*, the two sets of firing rates agree well with one another over a wide stimulus-intensity range, 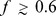, i.e., as long as the input to the sensory neurons is not too weak. For much of this regime, especially near 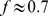, the model sensory neuron firing rates are typically between 20 Hz and 100 Hz, closely resembling experimentally observed firing rates of biologically realistic neurons, such as retinal ganglion cells [34], [46].

With the embedded linear network mapping (2), we arrive at our hypothesis that CS can potentially be a governing principle in transmitting sparse stimuli from the receptors to the sensory neurons, while simultaneously achieving a great reduction in the number of sensory neurons. In signal processing, the well-known Shannon-Nyquist theorem asserts that we must sample a signal with a given bandwidth uniformly at the rate of twice that bandwidth in order to be able to faithfully reproduce it [8]. However, according to CS theory, images that are (approximately) sparse in a wavenumber-space can be reconstructed from random samplings whose number 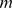 is much smaller than the number 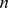 of pixels composing the image by finding the reconstruction with the smallest number of nonzero wavenumber-space components. Ref. [43] shows that this difficult optimization problem becomes equivalent to the much simpler question of finding the reconstruction whose wavenumber-space-component magnitudes add up to the smallest sum. Mathematically, one thus replaces a computationally expensive 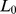 optimization problem in wavenumber space by a much simpler 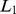 optimization problem, which can be efficiently solved via several optimization algorithms [47], [48].

By applying the CS approach of Candès and Tao to the linear mapping (2), we can reconstruct the stimulus from our model spike trains [7]. Thus, to estimate the sensing accuracy of our model early-sensory-pathway network, we measure the firing rates of each neuron in this network, and use the linear network mapping embedded in this model to carry out the relevant 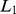 optimization procedure for finding the sparsest stimulus reconstruction. In particular, we reconstruct the stimulus given the rates 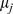, which we measure from the full simulation of the I&F network (1), by minimizing the sum 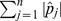, where 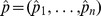 is the vectorization of the two-dimensional discrete cosine transform of the pixel matrix corresponding to stimulus 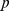, subject to the condition that the stimulus components 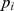 satisfy the linear system (2). (See the Methods Section for details.) It is important to remark that this reconstruction procedure requires only a brief simulation time, generally no more than 100 ms, since any initial transients in the network dynamics are very brief and typically last no more than 25 ms. In the next section, we further analyze the dependence of the CS reconstruction on the simulation time, and demonstrate that successful signal recovery takes place over a biologically realistic time-scale.

**Figure 3:**
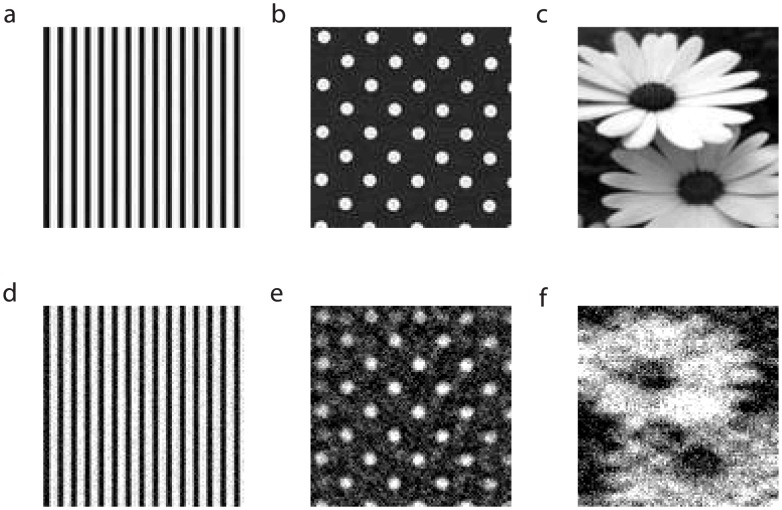
Stimulus reconstructions with CS. **a**, **b**, **c** two-dimensional images with 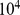 pixels, **d**, **e**, **f** reconstructions by CS; 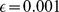, percent of Fourier components greater than 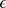 is 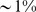. We choose 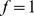 and 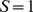, simulating network dynamics and recording neuronal spikes for a run-time of 200 ms.

We display three sets of results of our CS reconstruction procedure in Fig. 3, for which the stimuli are images of increasing complexity: stripes, dots, and flowers. Visually, the CS algorithm renders recognizable reconstructions of all the objects, and performs best with large shapes, flat surfaces, and gradual transitions, while leaving some graininess, which appears especially pronounced near sharp edges.

### Network Characteristics for Optimal Reconstruction

In determining the type of networks that can best take advantage of stimulus sparsity and optimally encode information, we study how the relative error, 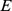, of the CS stimulus reconstruction depends on the various model network characteristics. We measure this error using the formula 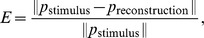
where the Euclidean norm, 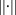, is defined analogously to the definition in the caption of Fig. 2. To isolate the effect of each characteristic, we vary only one parameter at a time while holding the remaining parameters constant.

**Figure 4:**
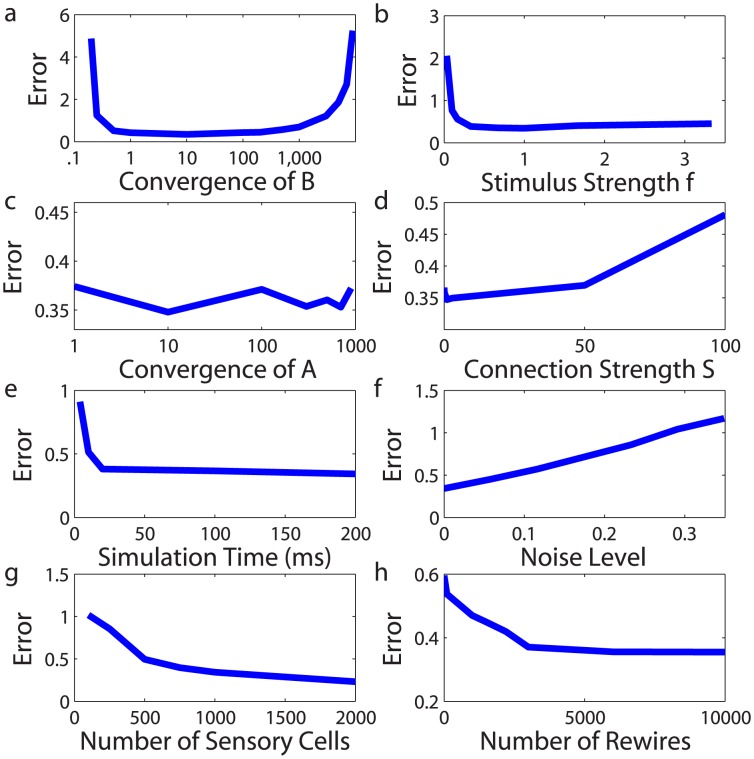
Optimal CS network characteristics. Characteristics of optimal CS networks examined using the dependence of relative reconstruction error on network features: **a** convergence of the sensory-cell (sensory-neuron) to receptor connection matrix 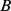, **b** stimulus strength 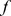, **c** convergence of the sensory-cell connection matrix 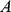, **d** connection strength 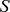, **e** simulation time over which spikes are recorded, **f** variance of Gaussian noise added to stimulus components, **g** number of sensory-cells, and **h** level of randomness in the sensory-cell to receptor connection matrix 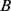. In panel **a**, we normalize the stimulus strength 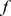 by 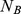, the number of nonzero entries in the receptor-to-sensory-neuron connectivity matrix 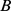, to keep the amount of drive to a sensory neuron approximately constant when changing convergence. The minimal relative error at the optimal convergence in panel **a** is approximately equal to 0.35.

First, we address how these CS networks depend on the convergence of the connections between the receptors and the sensory neurons, as represented by the matrix 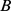 [49], [50]. As shown in Fig. 4a, the error decreases until the optimal convergence of about 10 is reached, and then increases. We remark that the high error for low convergence levels is due to the model sensory network not being able to sample all the receptors, while for high convergence levels all the sensory neurons receive nearly identical input. It should be clear why the convergence 10 is optimal; it is the ratio *n*∶*m* of the receptors versus the sensory neurons for our network model. At this ratio, with very high probability, each receptor feeds into precisely one sensory neuron. Due to the random sampling by the sensory neurons, on the other hand, again with high probability, the number of receptors relaying stimuli to a given sensory neuron will be approximately *n*∶*m*. Thus, all or most of the stimulus is used by the model sensory network, and there is little or no over- or undersampling.

Likewise, we address the question of how stimulus strength 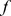 controls the optimality of CS networks. In Fig. 4b, we fix the convergence of 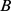 and the relative component sizes of the stimulus 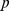, and scale linearly the overall stimulus strength 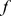. We observe that the reconstruction is best for moderate strength values, with particularly high reconstruction error for low 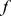, and slowly degrading reconstruction quality as 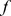 becomes too large. For the optimal stimulus strength, the sensory neurons are then neither underdriven, such that there are not enough firing events to properly encode network input, nor driven so hard that their interaction becomes too strong, overwhelming the information in the input signal.

In contrast, as displayed in Fig. 4c, the reconstruction error appears to depend little on the convergence of the matrix 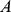 encoding the connections among the sensory neurons. In particular, for the error size, it makes little difference whether a sensory neuron receives many weak pulses or a few strong pulses from its neighbors, indicating that the amount of fluctuations received from within the sensory neuron network plays a rather small role. The error also appears to be relatively independent of the overall connection strength, 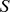, at sufficiently low 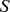-values, and then grows linearly with 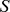, as shown in Fig. 4d. This reflects the fact that cross-talk among the sensory neurons that is too strong is likely to drown out the signal received from the receptors. Altogether, it thus appears that the connections among sensory neurons neither improve nor degrade the performance of the model network as long as their strengths are moderate. In the case of the retina, we note that while it was previously thought that there is no recurrent connectivity among retinal ganglion cells, recent experimental work shows that there is indeed gap-junction-type coupling among specific types of ganglion cells. [11], [33]–[35], [51], [52]. In either case, as long as the recurrent coupling is not too strong, the model sensory pathway will still accurately encode sparse stimuli. Therefore, the results of this work may more broadly apply to various types of ganglion cells, exhibiting diverse types of connectivity.

Clearly, for a CS network to be dynamically responsive in capturing transient stimuli, the system should be able to rapidly sample the stimulus within a sufficiently short time interval from the stimulus onset for the CS reconstruction. As shown in Fig. 4e, the reconstruction error drops precipitously until the sampling time increases to about 25 ms, and then remains approximately steady. The 25 ms time scale agrees with typical sensory time scales [53], [54]. To address the possibility of minor distortions of information along sensory pathways, we further address how the performance of a CS network is degraded in the presence of noise. As shown in Fig. 4f, we find that the relative reconstruction error grows approximately linearly with the variance of the Gaussian noise added to each stimulus component, demonstrating that a recognizable reconstruction is still achievable even in the presence of relatively high-variance noise.

Since thus far we have used a fixed number of sensory neurons, a natural question to ask is how the performance of a CS network improves as the number of sensory neurons increases. Fig. 4g shows that the performance will in fact improve with additional sensory neurons given a fixed number of receptors and corresponding optimal convergence. Since the reconstruction quality improves significantly less with the addition of sufficiently many sensory neurons, we observe that adding too many sensory neurons may be wasteful from a computational point of view, further justifying the optimality of sensory pathway architecture in processing sparse stimuli.

Hypothesizing that randomness is a key aspect in CS network sampling, we examine a central question of just how randomly sensory neurons need to sample the stimulus in order to achieve optimal sparsity encoding. To answer this question, we first design the connectivity matrix 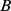 so that all sensory neurons sample receptors from a regular grid. Then, we sequentially remove an original connection in 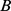, and replace it by a connection between a randomly chosen receptor and the same sensory neuron. (See Methods Section for details.) From Fig. 4h, we see that the error decreases rapidly until 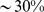 of the initial regular connections have been rewired, and then slowly levels off. Therefore, some degree of randomness is in fact necessary for a viable reconstruction, however the sampling need not be completely random for successful sparsity encoding. In fact, the success of intermediate levels of randomness may help to explain how the localized sampling in receptive fields further improves the performance of the CS network, which we will address later in this section.

**Figure 5:**
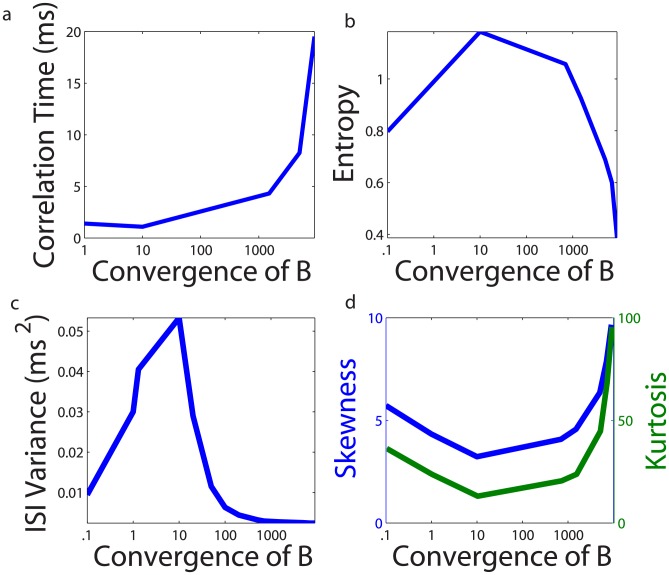
CS network dynamics for optimal signal encoding. Impact of the convergence of the sensory-cell to receptor connection matrix, 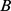, on local-field-potential (“LFP”) and spike-train statistics: **a** correlation time of the sensory-cell network “LFP”, **b** entropy of the sensory-cell network interspike-interval (ISI) histogram, **c** variance of the sensory-cell network ISI distribution, and **d** skewness and kurtosis of the sensory-cell network ISI distribution.

Next, we investigate the characteristics of sensory-neuron spike dynamics that are significant in these sparsity-encoding CS networks. We find that the parameter regimes yielding the least error in the stimulus reconstructions are those in which the largest degree of variability or disorder exists among the dynamics of the model sensory neurons. We compute the average sensory network membrane potential, 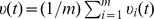, which roughly models the network local field potential (“LFP”) signal measured experimentally [55], [56], to give an indication of the variability in network dynamics. In Fig. 5a, we plot the “LFP” correlation time as a function of the convergence of the receptor-sensory-neuron connection matrix 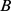. It is clear that the “LFP” decorrelates the fastest at the optimal convergence value, indicating relatively aperiodic network dynamics. To quantify the corresponding network information content, we compute the entropy, 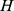, of the spike train produced by the network of neurons 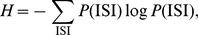
where 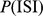 denotes the probability distribution of the interspike-interval (ISI) lengths, computed from a binned histogram of ISI's collected from each sensory neuron in the network. In our case, the entropy of the ISIs measures the spike-train information capacity, and therefore gives an indication as to how much possible information can be encoded by the sensory-neuron network over the time-scale of network activity [57], [58]. This entropy reaches its maximum at the optimal convergence, as displayed in Fig. 5b, thereby transmitting the maximum amount of information. It is important to remark that while we specifically use firing rates to reconstruct stimuli, information about the actual sensory-neuron spike trains is embedded in the firing rate statistics. Since the firing rate gives the lowest order of information regarding the ISI distribution, the ISI distribution is of particular interest in quantifying the information encoded by sensory-neuron activity.

In examining the distribution of the ISI's, we observe a rich firing structure among the sensory neurons at the optimal convergence of connectivity matrix 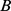. We demonstrate in Fig. 5c how the variance 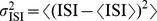 of the ISI distribution depends on the convergence of 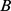. (Here, 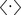 denotes the mean over the distribution.) This variance is clearly maximal at the optimal convergence. Moreover, the ISI structure is further characterized by its near-Gaussian distribution at optimal convergence value, as shown in Fig. 5d, reaching its minimal skewness, 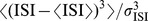, and kurtosis, 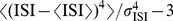, which vanish for the Gaussian distribution. From these observations, it is clear that the connectivity between the receptors and sensory neurons plays a large role in determining the information content of the sensory neuron spike dynamics, and by maximizing the information content of these spikes, stimuli may be optimally encoded.

### Biological Extensions

**Figure 6:**
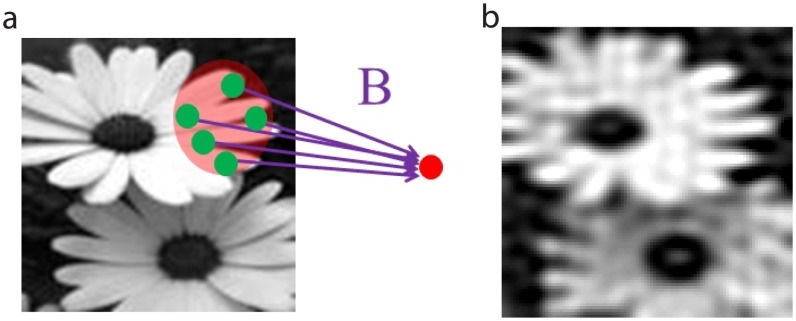
Localized receptive fields. **a** Stimulus sampling via localized receptive fields. **b** The corresponding CS stimulus reconstruction.

We further corroborate the hypothesis that evolution may have driven early sensory pathways to become CS networks by incorporating a biologically realistic feature, i.e., localized receptive fields, into our model CS network. We discover that this feature indeed improves the performance of the highly idealized CS network we have investigated so far. We model such a receptive field by using a variant of the model in which each sensory neuron samples receptors primarily within a small area, which is closer to biological realism than random sampling [18], [19]. In particular, if the coordinates of the pixel representing a receptor are given by the vector 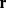 and the coordinates of the receptive-field center corresponding to a chosen sensory neuron are given by the vector 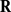, we take the probability that a connection will exist between the two as 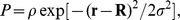
where 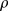 represents the probability of a connection if 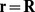, and 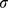 is the size of the sensory neuron's receptive field in the units of pixel size. A schematic illustration of this type of sampling is depicted in Fig. 6a, with the parameter values, 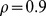 and 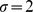, resulting in a convergence of 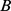 of 25. A reconstruction of the original image from the firing rates produced by this model is shown in Fig. 6b. Note that we found the error of this reconstruction to be 0.19, which is much less than the error of 0.35 we obtained for completely random stimulus-sampling over the entire receptor pool, shown in Fig. 4a. This result, reaching beyond the conventional CS theory, underscores the importance of the local-receptive-field architecture in the evolution of the CS properties of sensory pathways.

**Figure 7:**
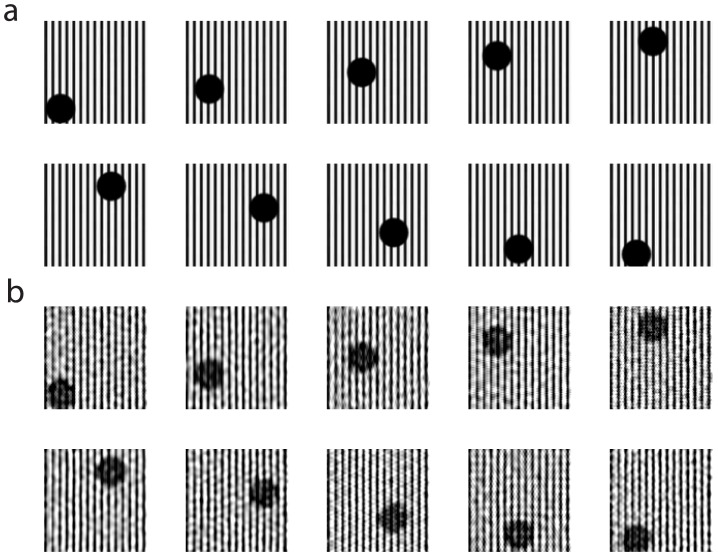
Moving stimulus reconstruction. **a** A moving stimulus and **b** its reconstruction. Localized receptive fields were used; the average relative error over the frames is 0.24. Image frames were presented for 25 ms each.

We remark that we can also reconstruct moving stimuli using our CS approach. The reconstruction of a ten-snapshot image sequence spaced 25 ms apart is displayed in Fig. 7. In reconstructing each image frame, we only use spikes counted during the time-course over which each respective image is presented. In this way, we consider ten CS recovery problems, with each corresponding to a separate set of observed firing rates. From the highly accurate reconstructions even in the case of moving stimuli, it is clear that the CS architecture is therefore feasible for natural environments in which stimuli are constantly in motion. Moreover, if the image frames instead change every 200 ms, the average reconstruction quality is improved further. As in the case of realistic retinal video processing, correlations between frames and close corresponding dynamical regimes therefore allow for rapid encoding of changing stimuli [59]–[61].

## Discussion

We hypothesize that the CS principle for sparse-stimulus transmission in neuronal networks, as demonstrated in our computational model, should also hold in real neuronal systems in the brain. In more general settings, the underlying linear structure could be recovered using the first-order Wiener kernel from non-linear systems analysis for the entire network [62], [63]. Similarly, in deriving an input-output relationship outside of the mean-driven regime, a linear-nonlinear (LN) model can also be developed through application of a linear spatiotemporal filter and a static nonlinear transformation (e.g. sigmoidal function), which often can be cast in a linear form if the inverse of the nonlinear transformation exists [64]. In either case, once the underlying linear structure is discovered, the presented methodology could in principle be used to attempt to reconstruct sparse stimuli using very few neuronal output measurements.

Mathematically, this work suggests two important extensions to conventional CS theory. First, while compressed sensing is traditionally applied to static linear systems, we demonstrate one possible way of generalizing this theory to dynamical systems that model a large number of interacting agents. Second, the improvement in stimulus encoding yielded by localized random sampling akin to receptive fields suggests that alternative sampling schemes, aside from purely random sampling, may in fact yield better reconstructions so long as there is a sufficient degree of incoherence in the samples such that CS is applicable. From this standpoint, measurement devices engineered with localized random sampling may be able to more successfully encode signals than by applying the completely random sampling conventionally used in compressive sensing data acquisition [65]. Likewise, engineered devices sampling the output of a time-evolving network may also have the capacity to reconstruct network input using compressive sensing combined with an underlying linear input-output network structure similar to the neuronal network studied in this work.

Finally, we point out a new way of looking at the mathematical framework of sparsity. Our findings give rise to a network definition of stimulus sparsity, freeing this concept from any dependence on the particular choice of wavenumber-space or other component-space transform as in conventional definitions of sparsity. In particular, we can define a stimulus as sparse if it can be accurately and efficiently transmitted through a sensory-pathway-type network, such as one that allows for a significant reduction in the numbers of downstream sensory neurons versus the numbers of upstream receptors. This alternative definition of sparsity therefore directly relates the structure of a stimulus to the type of network dynamics it evokes. Rather than indicating sparsity by the number of non-zero signal components, sparsity can alternatively be determined in the network framework by the amount of stimulus information embedded in the evoked network dynamics. Thus, visual images are clearly sparse according to both the networks that sample them completely randomly and those with localized receptive fields.

In the long way towards understanding how the brain forms a specific percept from a given stimulus, one must first understand how the brain samples this stimulus. Our aim here was to examine the hypothesis that the CS principle has evolved to govern the information transduction and retention of sparse stimuli in a sensory pathway while achieving a great reduction in the number of sensory neurons. Our work shows that this hypothesis indeed successfully captures information propagation in our model sensory network. In particular, our results on these network characteristics may provide insight into the CS properties of corresponding networks in the brain.

## Materials and Methods

### 1 Correlation Time Definition

The correlation time gives the expected amount of time necessary for signal responses to become decorrelated and is defined mathematically as 
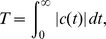
where 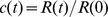 is the correlation function of the “LFP” 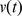, with 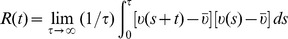
and time average of the “LFP” is 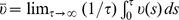. A short correlation time implies less periodicity and therefore greater variability in the “LFP”.

### 2 Compressed Sensing Reconstruction

To reconstruct a stimulus, 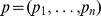, from the sensory-neuron firing rates, 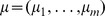, we first cast the linearized firing-rate model (2) into a form to which compressed sensing may be applied. To apply compressed sensing in recovering a sparse representation of 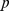, we consider the vectorization of the two-dimensional discrete cosine transform of the stimulus pixel matrix, 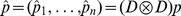, where 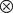 denotes the 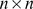 Kronecker product 
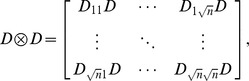


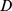
 is the 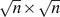, one-dimensional discrete cosine transform matrix with entries 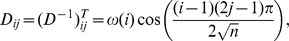


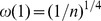
, and 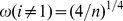. In solving the related problem of recovering 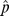, the linear model we consider is 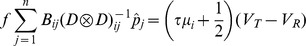


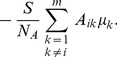
(3)


Since the cosine transform of the stimulus, 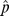, is sparse and the matrix 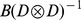 is random, recovering 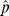 is reduced to minimizing the sum 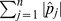 [6], [7] under the constraint (3). Solving this minimization problem is equivalent to solving the well-known 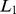 optimization problem 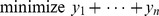


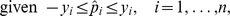
under the constraint (3). We solve this optimization problem with a greedy algorithm, known as the Orthogonal Matching Pursuit [48]. Once 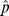 is recovered, we recover the stimulus 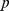 using the inverted two-dimensional discrete cosine transform of the matrix representation of 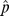.

### 3 Regular versus Random Sampling

As mentioned in the main body of this paper, CS theory posits that random sampling of sparse images significantly reduces the sampling rate as compared to uniform sampling of finite-bandwidth stimuli, while yielding the same quality of the reproduction. In particular, for uniform sampling, the Shannon-Nyquist theorem requires that finite-bandwidth stimuli must be sampled at the rate of twice their bandwidth in order to achieve a faithful reconstruction [8]. In our case, this would mean sampling by 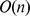 sensory neurons when all the frequencies are represented in the image used as the stimulus. This is because we need to capture each Fourier mode represented in the stimulus in at least two points. On the other hand, the compressed-sensing theory implies that much less frequent sampling should suffice for 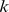-sparse stimuli to perfectly reconstruct the stimulus with probability one, in particular, on the order of 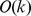 [6], [7], provided the sampling is sufficiently random. Again, this is because, with probability one, we will thus capture each represented Fourier mode in two points. This is certainly not true if we sample the stimulus on a regular, coarse grid with 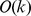 points in the spirit of the Shannon-Nyquist theorem, unless the stimulus contains nothing but the lowest 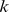 modes. In fact, such sampling diminishes the resolution. We here elaborate on the illustration of this fact, as depicted in Fig. 4h.

In regularly sampling the stimulus, 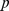, sensory neurons sample only receptors that lay on a coarse grid contained within the finer grid of receptors, modeled by the 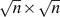 pixel matrix representation of 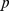. The coarse regular grid, say of size 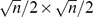, is composed of all even-numbered row and column entries of the finer pixel matrix. Fixing convergence at its optimal level, the regular sampling scheme randomly connects sensory neurons to receptors on the coarse grid. To add more randomness and a larger variety of frequency modes to the sampling scheme, we randomly select sensory neurons connected to receptors on the regular grid, and then randomly rewire them with any of the receptors on the pixel matrix.

As displayed in Fig. 4h, even if the sampling is random on a coarse grid, not enough frequency modes may be captured to yield a faithful signal reproduction. Upon random rewirings to the finer grid of receptors, more frequency components may be detected, thereby improving the quality of the reconstruction. However, once the sampling scheme is sufficiently random and enough variety in frequency modes is captured, an accurate reconstruction can be achieved with little improvement following additional rewirings.
